# Elevated Aggression and Reduced White Matter Integrity in Mild Traumatic Brain Injury: A DTI Study

**DOI:** 10.3389/fnbeh.2018.00118

**Published:** 2018-06-27

**Authors:** Natalie S. Dailey, Ryan Smith, Sahil Bajaj, Anna Alkozei, Melissa K. Gottschlich, Adam C. Raikes, Brieann C. Satterfield, William D. S. Killgore

**Affiliations:** Social, Cognitive and Affective Neuroscience Laboratory, Department of Psychiatry, College of Medicine, University of Arizona, Tucson, AZ, United States

**Keywords:** mild traumatic brain injury, aggression, white matter integrity, diffusion tensor imaging, corpus callosum, post-concussive symptoms

## Abstract

Mild traumatic brain injury (mTBI) remains the most commonly reported head injury in the United States, and is associated with a wide range of post-concussive symptoms including physical, cognitive and affective impairments. Elevated aggression has been documented in mTBI; however, the neural mechanisms associated with aggression at the chronic stage of recovery remain poorly understood. In the present study, we investigated the association between white matter integrity and aggression in mTBI using diffusion tensor imaging (DTI). Twenty-six age-matched adults participated in the study, including 16 healthy controls (HCs) and 10 individuals in the chronic stage of recovery (either 6-months or 12 months post-mTBI). Psychological measures of aggression included the Buss-Perry Aggression Questionnaire and the Personality Assessment Inventory (PAI). Axonal pathways implicated in affective processing were studied, including the corpus callosum, anterior thalamic radiation, cingulum and uncinate fasciculus, and measures of white matter integrity included fractional anisotropy (FA), mean diffusivity (MD), radial diffusivity (RD) and axial diffusivity (AD). We found that adults with mTBI in the chronic stage of recovery had higher levels aggression. Individuals with mTBI also had greater RD in the corpus callosum compared to HCs, indicating reduced fiber integrity. Furthermore, we observed a significant association between reduced white matter integrity in the corpus callosum and greater aggression. Our findings provide additional evidence for underlying neuroanatomical mechanisms of aggression, although future research will be necessary to characterize the specific relationship between aggression and the white matter pathways we identified.

## Introduction

Mild traumatic brain injury (mTBI) accounts for roughly 75% of the 1.5 million head injuries reported annually in the United States (Centers for Disease Control and Prevention, 2003). However, not all individuals who sustain a mTBI seek medical treatment, making it difficult to identify and track post-concussive symptomology and long-term disabilities in this population. During the acute and subacute phases of recovery, symptoms generally fall into one of three categories, including physical (i.e., headaches, dizziness and sleep disruptions), cognitive (i.e., impaired attention and memory, reduced processing speed and poor concentration) and affective (i.e., increased irritability, anxiety and depression) disruptions (Prince and Bruhns, 2017). Furthermore, aggression is one of the most common affective symptoms, with upwards of 40% of individuals reporting increased aggression, hostility, or irritability after sustaining a mTBI (Kim et al., 1999; Bailie et al., 2015; Epstein et al., 2016; Roy et al., 2017).

One possible avenue through which to gain a better understanding of aggression in mTBI is to examine acquired pathology within the neural systems that contribute to affective/emotional behavior. Emotional episodes are believed to arise from interactions between cognitive, physiological and behavioral processes, such that bodily/behavioral reactions are first initiated in response to a situation (and its cognitive interpretation), and these reactions are then perceived and interpreted at multiple levels—leading to the behavioral expression, experience, recognition and subsequent regulation of the elicited emotion (Lindquist et al., 2012; Barrett and Satpute, 2013; Smith and Lane, 2015; Smith et al., 2017). Several large-scale neural networks have been implicated in the emotion-related processes described above, with contributing regions within distinct prefrontal, cingulate and subcortical areas (Barrett and Satpute, 2013). As individuals with a history of mTBI have been found to exhibit problems with the experience, expression and control of anger, this suggests that such individuals may exhibit pathology in these emotion-related processes, and within the neural networks that appear to implement them (Bailie et al., 2015). However, the potential neural mechanisms that underlie the expression and regulation of anger/aggression, and the relationship between neuronal injury sustained during mTBI and affective dysregulation, have not yet been thoroughly examined.

Of the many interconnected large-scale neural networks embedded within association cortices, white matter pathways connecting the temporal lobe, amygdala and orbitofrontal, medial prefrontal, and cingulate cortices are believed to play especially important roles in the emotion-related processes mentioned above (Barrett and Satpute, 2013). One such pathway is the anterior thalamic radiation, a fiber bundle connecting the thalamus, orbitofrontal cortex and anterior cingulate cortex, which is believed to be involved in affective response generation/regulation. Damage to this pathway is associated with emotional dysfunction (e.g., depression) and reduced self-awareness of emotion in clinical populations (Sussmann et al., 2009; Kubota et al., 2012). Another important pathway is the uncinate fasciculus, which connects the anterior temporal lobe, amygdala and orbitofrontal cortex. This pathway may facilitate top-down prefrontal modulation of the amygdala, which is believed to be important for promoting context-appropriate affective responses (Gershman et al., 2013; Chan et al., 2016). Reductions in white matter integrity within the uncinate fasciculus have also been found in populations that exhibit patterns of aggression and/or deficits in emotional regulation, including adults with multiple concussions (Goswami et al., 2016) and specific psychiatric disorders (Sundram et al., 2012). Yet another relevant pathway is the cingulum, which contains fibers connecting medial prefrontal, cingulate, and medial parietal regions. These regions support a network often termed the “default network”, and is thought to play an important role in the conceptualization of affective states through the integration of prior experiences (Binder et al., 2009). Finally, the corpus callosum connects left and right hemisphere components of a range of cortical systems, including those subserving motor, perceptual, and cognitive functions. Furthermore, there is some evidence that interhemispheric signal transfer through the corpus callosum may play an integral role in cognitive processes that contribute to aggressive behavior (Schutter and Harmon-Jones, 2013).

When considering acquired neural network pathology in mTBI, time since injury and injury severity are likely key factors for understanding affective symptomology. Neurobehavioral symptoms are often dynamic in the early stages of injury, with the majority of individuals recovering quickly from mTBI (Binder et al., 1997; McCrea et al., 2003). However, there is evidence to suggest that a significant proportion of individuals experience persistent and potentially disruptive symptoms months and years after their injury (see Ruff, 2005). Findings related to affective disruption are also inconsistent in the few studies that directly investigate mTBI-related aggression. For example, some studies report diminished irritability and/or anger with increased time since injury (Kim et al., 1999; Bailie et al., 2015), suggesting improvements in neurobehavioral symptoms over time. In contrast, Baguley et al. (2006) found that the prevalence of aggression was similar at 6, 24 and 60 months post-injury, while another study by Roy et al. (2017) documented elevated aggression 6 months to 1-year post-injury—suggesting aggression is a persistent symptom associated with brain injury. Moreover, individuals who experience persistent symptoms, including affective disruption, may be prime candidates for clinical intervention. Similar discrepancies have been found regarding injury severity. An inverse relationship between injury severity and aggression has been documented, where individuals with mild to moderate TBI were more likely to report post-injury irritability/aggression (Kim et al., 1999). However, other studies report no significant relationship between injury severity and aggression (Rao et al., 2009; Bailie et al., 2015). The use of mixed samples (e.g., time since injury and/or injury severity) may account for the discrepancies in the current literature and complicate the interpretations regarding mTBI-related aggression.

The manifestation and duration of mTBI-related aggression beyond the acute and subacute stages of recovery is not well understood, nor are the underlying neural mechanisms. Given the inconsistencies in the literature, the present study focused exclusively on individuals in the chronic phase of recovery (6-months and 12-months post-injury) and with a TBI that was classified as mild. The purpose of the present study was twofold: (1) to assess chronic post-concussive aggression in those with mTBI; and (2) to identify fiber pathways associated with aggression. We hypothesized that adults with mTBI would exhibit elevated levels of aggression relative to healthy controls (HCs). Using neural models of emotion to guide the selection of targeted white matter pathways, white matter integrity of the corpus callosum, cingulate, anterior thalamic radiation and uncinate fasciculus was hypothesized to show reduced integrity in the mTBI population, and these white matter integrity reductions were predicted to show associations with aggression.

## Materials and Methods

### Participants

Twenty-six age-matched young adults were enrolled in the present study, including 16 HCs and 10 individuals with mTBI; three at 6-months post-injury and seven at 12-months post-injury. Eligibility criteria required participants to be between 18 years and 45 years of age, native English speakers and right handed. For those in the chronic mTBI group, brain injury documentation from a doctor, physician, or other qualified witness to the injury was required prior to enrollment in the study. Severity was classified as mild based on the ACRM and the Department of Veterans Affairs, Department of Defense (2016), where mTBI was defined as a physiological disruption of brain function resulting in temporary loss of consciousness (<30 min), transient posttraumatic amnesia (<24 h), altered mental state (i.e., feeling dazed, disoriented, or confused), and/or focal neurological damage that may or may not be transient (American Congress of Rehabilitation Medicine, 1993). Exclusionary criteria included: (1) a history of psychiatric or neurological disease; (2) pregnancy; (3) previous or ongoing alcoholism or substance abuse; (4) more than three TBIs in a lifetime; or (5) contraindication to MRI. In the present sample, mTBI was the result of sports related injuries (70%), vehicular accidents (20%) and falls (10%). Participants in this study are part of a larger ongoing study, investigating neuropsychological function across multiple stages of recovery from mTBI. The current study was approved by the Institutional Review Board at the University of Arizona and the U.S. Army Human Research Protections Office (HRPO), and all participants provided written informed consent in accordance with the Declaration of Helsinki.

### Neuropsychological Assessments

Participants completed a battery of neuropsychological assessments on the same day and prior to the collection of neuroimaging data. Paper and pencil assessments were administered by a trained full-time research technician in a quiet testing room located in the laboratory.

#### Wechsler Abbreviated Scale of Intelligence Test (WASI-II)

All participants completed the Wechsler Abbreviated Scale of Intelligence test (WASI-II; Wechsler, 1999). The WASI-II is highly correlated (*r* = 0.92) with the longer Wechsler Adult Intelligence Scale (WAIS; Wechsler, 2008) and was used to obtain a measure of general intellectual ability or “IQ”. Full Scale IQ was used to assess overall intelligence and ensured participants enrolled in the study exhibited cognitive functioning within normal limits.

#### Buss-Perry Aggression Questionnaire

Aggression was measured using two different questionnaires. The Buss-Perry Aggression Questionnaire (BPAQ; Buss and Perry, 1992) consists of 29 items, rated on a 5-point scale from “extremely uncharacteristic” to “extremely characteristic”. The BPAQ provides an overall measure of aggression (total aggression) and four subscales including physical aggression, verbal aggression, anger and hostility. BPAQ-Physical measures one’s tendency to use threats, and/or physical harm towards others and objects. BPAQ-Verbal measures disagreements and argumentative behavior, while BPAQ-Anger assesses irritability and control over one’s temper. Finally, BPAQ-Hostility refers to feelings of jealousy, suspicion and resentment. Moderate to high reliability has been established for the BPAQ (Harris, 1997) and participants were given as much time as needed to complete the assessment.

#### Personality Assessment Inventory

The personality assessment inventory (PAI; Morey, 1991) was the second self-report measure of aggression. The PAI was administered on the computer and required roughly 45 min to complete. This 344-item inventory uses a four-alternative scale ranging from “Totally False” to “Very True”, to assess 22 non-overlapping scales, including aggression. Total aggression on the PAI measures characteristics and attitudes related to anger, assertiveness and hostility. The PAI has three subscales which include aggressive attitude (hostility and poor control over anger), verbal aggression (assertiveness and readiness to express anger to others), and physical aggression (tendency to be involved in physical altercations). The PAI has been found to be a valid and clinically useful measure of psychiatric and emotional disturbances in adults with TBI (Till et al., 2009). Due to time restrictions and computer error, PAI scores were incomplete for one HC and three mTBI participants.

#### Beck Depression Inventory

Given the strong association between depression and aggression (Rapoport et al., 2003), the Beck Depression Inventory (BDI-II; Beck et al., 1961) was used to assess post-injury depression. The BDI-II has been shown to discriminate well between clinical and non-clinical populations, where a score of 13 or greater is indicative of mild clinical depression (Lasa et al., 2000). Participants scored the 21-item inventory using a 4-point scale ranging in severity from 0 to 3 for each item. Clinical levels of depression have been shown to be a comorbid symptom of mTBI (Jorge et al., 2004; Baguley et al., 2006), therefore, BDI-II scores were used as a covariate in subsequent analyses, allowing for the comparison of aggressive tendencies while controlling for behaviors associated with depression.

### Neuroimaging

Magnetic resonance imaging (MRI) data were collected at the University of Arizona, using a whole-body Siemens Skyra 3.0 Tesla with 32-channel head coil (MAGNETO Skyra Siemens Healthcare). Diffusion weighted data were acquired using single-shot echo planar imaging (TR = 9600; TE = 88; acquisition matrix = 128 × 128; FOV: 256 × 256; slice thickness = 2 mm, no gap). Diffusion gradients were applied along 72 directions, with b = 1000 s/mm^2^ and six non-diffusion weighted images (b_0_). A preprocessing pipeline consisted of artifact and head motion correction using TOPUP eddy in the FMRIB Software Library (FSL; Andersson and Sotiropoulos, 2016). The FMRIB Diffusion Toolbox was used for brain extraction (Smith, 2002), and fitting of the diffusion tensor model (DTIFIT; Behrens et al., 2003). DTIFIT calculates fractional anisotropy (FA) and mean diffusivity (MD), while axial diffusivity (AD) and radial diffusivity (RD) were calculated from DTIFIT outputs using the following formulas:

(1) $$AD=\lambda_{1}$$

(2) $$RD=(\lambda_{2}+\lambda_{3})/2$$

Measured differences in anisotropy can result from axonal density and/or myelination, where AD and RD have been associated with axonal integrity and demyelination, respectively (Song et al., 2002, 2005). Thus, these metrics allow for the quantification of fiber pathway integrity, and may provide reliable biomarkers of mTBI-related symptoms. Tract-Based Spatial Statistics (TBSS; Smith et al., 2006) was used for nonlinear registration to a standard template (FMRIB-58) and affine-aligned to 1 × 1 × 1 mm Montreal Neurological Institute (MNI) space using FNIRT (de Groot et al., 2013). Individually aligned images were then merged to create a single 4D image for each DTI metric, resulting in a total of four separate 4D images. White matter was identified using whole-brain skeletonized masks with a threshold of 0.20, a process that reduces voxels in the periphery where inter-subject variability or partial volume effects tend to be high.

### Targeted White Matter Tracts

**Figure 1:**
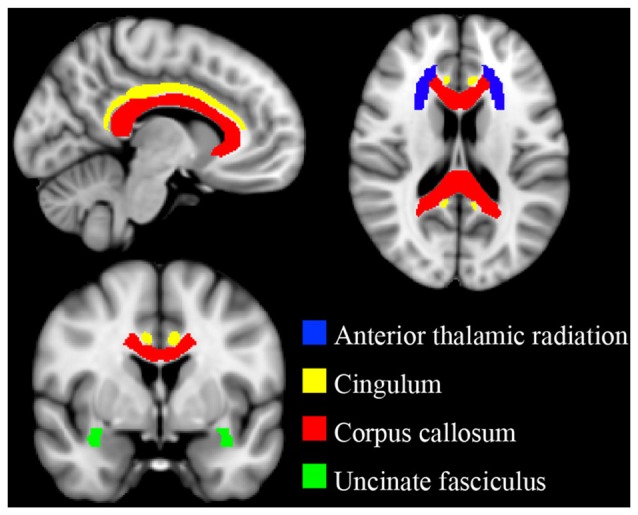
Targeted white matter tracts. Binary template masks from the ICBM-DTI-81 atlas were used to delineate white matter pathways of interest. Anterior thalamic radiation (blue), cingulum (yellow), corpus callosum (red) and uncinate fasciculus (green). Template masks are overlaid on a standard T1 montreal neurological institute (MNI) template (1 × 1 × 1 mm).

**Figure 2:**
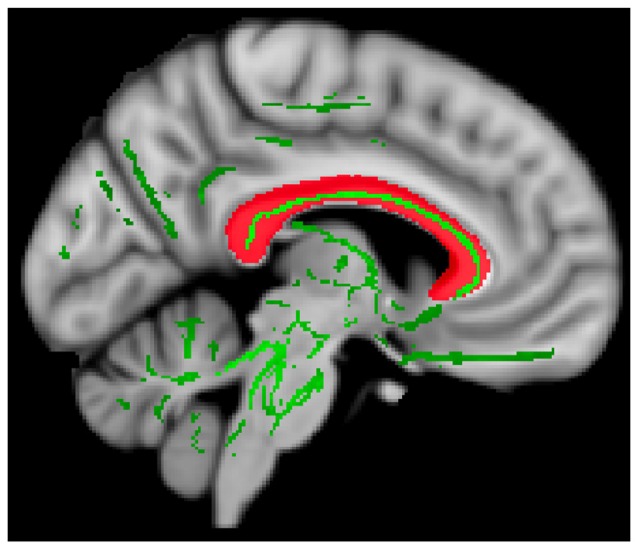
Extracting diffusion values. Data from a representative participant showing whole-brain 4D skeletonized data (green) overlaid on the mask of the corpus callosum (red). Only voxels from 4D data (green) overlapping with white matter mask (red) were extracted and included in subsequent analyses.

Binary template masks were created to extract voxels from targeted pathways including the corpus callosum, and bilateral cingulate, uncinate fasciculus and anterior thalamic radiation. Template masks were based on the ICBM-DTI-81 white-matter labels atlas overlaid on a standard template in MNI space (MNI_152_T1 1 × 1 × 1 mm; see Figure 1). The population-based atlas includes 48 labeled tracts from hand-segmented white matter parcellation maps based on averaged tensor maps derived from 81 subjects (Mori et al., 2008). Voxels from the 4D skeletonized images which overlapped a given white matter mask were included in subsequent analyses for that tract (see Figure 2). White matter integrity was quantified using diffusion and anisotropy properties, resulting in FA, MD, RD and AD values for all targeted tracts. Mean values for a given tract were obtained by averaging all values from voxels extracted from the white matter mask. By extracting values from the skeletonized data and targeting specific tracts of interest, this DTI-approach eliminates superfluous comparisons and provides higher detection power of white matter differences between the two groups.

### Statistical Analysis

Analyses were conducted using IBM SPSS Statistics (version 24.0). Independent sample *t*-tests were performed to identify group differences on continuous demographic measures, and a chi-square was used to test that group and gender were independent. General linear models (GLMs) were used to test whether the two groups differed on measures of aggression and DTI metrics, while controlling for potential effects of age, gender and depression (i.e., covariates). Group was entered into each GLM as a categorical independent variable, where the HCs was coded as “0” and individuals with mTBI were coded as “1”.

To test our first hypothesis addressing aggression in chronic mTBI, we fit individual GLMs with group as a categorical independent variable and BPAQ and PAI total aggression as dependent variables. Age, gender and depression were entered into each model as covariates. Based on significant findings, *post hoc* analyses were conducted to determine which aggression subscales contributed significantly to the overall between-group effect. Separate GLMs were calculated, with group as the independent variable and BPAQ or PAI subscales as the dependent variables, controlling for age, gender and depression.

To test our second hypothesis addressing group differences in the microstructure of targeted fiber pathways, individual GLMs were fit for each tract in the left and right hemisphere separately, with group as a categorical independent variable and DTI metrics (FA, MD, AD and RD) as the dependent variables, while accounting for the effects of age, gender and depression. False discovery rate (FDR; α = 0.05) was used to minimize Type I error associated with multiple comparisons (Benjamini and Yekutieli, 2001). For comparisons of microstructure integrity, pathways of interest were selected *a priori* and FDR-correction was adjusted within tract. Finally, Pearson’s partial correlations were calculated to quantify the unique relationship between white matter integrity and aggression. Given our interest in determining whether a relationship exists between white matter integrity and aggression, we computed these partial correlations within our whole sample, without stratifying by group. Partial correlation covariates included age, gender and depression and the correlations were restricted to overall aggression measures and tracts which showed significant between-group differences in the previous analyses.

## Results

### Neuropsychological

**Figure 3:**
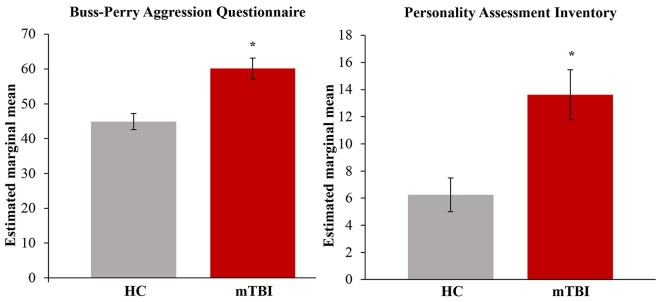
Total aggression by group. Significantly higher aggression on the Buss-Perry Aggression Questionnaire (BPAQ) and Personality Assessment Inventory (PAI) was found in those with mild traumatic brain injury (mTBI). Estimated marginal means represent the group means after adjusting for covariates in the model (age, gender and depression). HC, healthy controls; mTBI, mild traumatic brain injury. Error bars represent standard error of the mean. *p < 0.05; FDR-corrected.

Demographic characteristics are summarized in Table 1. Adults with mTBI reported significantly higher levels of aggression on the BPAQ (*F*_(1,21)_ = 13.22, *p* < 0.05; *η*^2^ = 0.39; FDR-corrected) and the PAI (*F*_(1,17)_ = 10.86, *p* < 0.05; *η*^2^ = 0.39; FDR-corrected), as compared to HCs (see Figure 3). In addition, BPAQ scores differed based on gender (*F*_(1,21)_ = 8.28, *p* < 0.01; *η*^2^ = 0.28).

**Table 1:** Demographic characteristics by group.

	Healthy Controls (*n* = 16)	Chronic mTBI (*n* = 10)	Statistic	*p*-value
Age, in years	22.69 (3.40)	22.40 (6.38)	*t*_(24)_ = 0.15	0.88
Gender – %female	50%	70%	$\chi_{\left(1\right)}^{2}$ = 1.01	0.43
Education, in years	14.19 (2.43)	12.80 (1.55)	*t*_(24)_ = 1.61	0.12
WASI-II Full-Scale IQ	111.31 (9.69)	111.90 (12.90)	*t*_(24)_ = −0.12	0.90
BDI	2.50 (3.08)	6.10 (7.64)	*t*_(24)_ = −1.69	0.10
Time Since Injury, in days				
6-months (*n* = 3)		184.67 (2.08)		
12-months (*n* = 7)		363.57 (2.99)		

*Note: Values are Mean (Standard Deviation), unless otherwise noted. mTBI, mild traumatic brain injury; WASI-II, Wechsler Abbreviated Scale of Intelligence – 2nd Edition; BDI, Beck Depression Inventory*.

**Figure 4:**
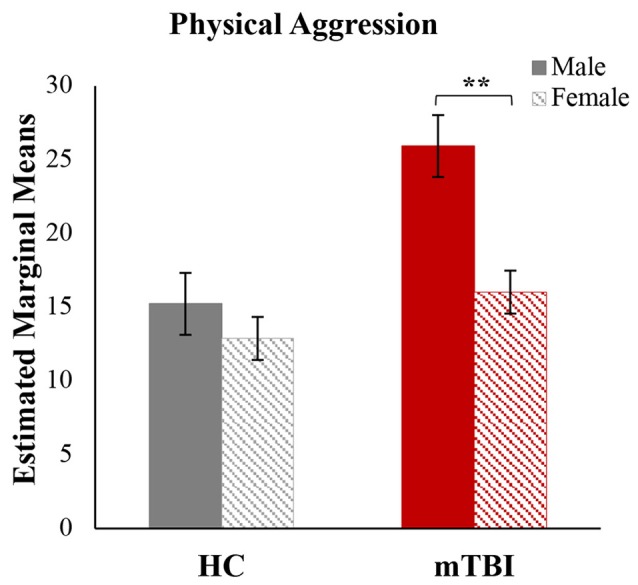
Physical aggression by group and gender. A significant group by gender interaction was found for physical aggression. Estimated marginal means for the physical aggression subscale of the Buss-Perry Aggression Questionnaire, after adjusting for covariates (age and depression). HC, healthy controls; mTBI, mild traumatic brain injury. Error bars represent standard error of the mean. **p < 0.01.

To better understand the driving factors associated with elevated aggression, *post hoc* analyses were conducted separately for BPAQ and PAI subscales (see Table 2). *post hoc* results were FDR-corrected at *p* < 0.05, within test. Adults with mTBI reported significantly higher aggressive attitude on the PAI and significantly higher levels of physical aggression and anger on the BPAQ. In the GLM for the BPAQ, gender was significantly associated with physical aggression (*F*_(1,21)_ = 9.28, *p* < 0.01; *η*^2^ = 0.31). Follow-up analyses were conducted to further explore these findings. An analysis of covariance was calculated with physical aggression as the dependent variable, group and gender as the independent variables, with age and depression as covariates. There was a significant main effect of group (*F*_(1,20)_ = 19.08, *p* < 0.001; *η*^2^ = 0.49), a significant main effect of gender (*F*_(1,20)_ = 15.64, *p* < 0.001; *η*^2^ = 0.44), and a significant group × gender interaction (*F*_(1,20)_ = 5.49, *p* < 0.05; *η*^2^ = 0.22). Figure 4 shows the *post hoc* results, in that males with mTBI reported significantly higher physical aggression (*M* = 25.67; *SD* = 4.04) than females with mTBI (*M* = 15.86; *SD* = 3.13), a result not observed in HCs.

**Table 2:** *Post hoc* group comparisons on aggression subscales.

	Healthy Controls (*n* = 16)	Chronic mTBI (*n* = 10)	*F-statistic*^a,b^	*p*-value	*Partial* η^2‡^
BPAQ					
Physical Aggression	14.19 (3.73)	18.80 (5.71)	13.22^a^	0.01*	0.39
Verbal Aggression	10.69 (2.92)	12.40 (4.01)	2.39^a^	0.14	0.10
Anger	9.75 (1.98)	12.20 (2.97)	9.44^a^	0.01*	0.31
Hostility	11.63 (4.02)	14.60 (4.38)	0.25^a^	0.05	0.17
PAI					
Aggressive Attitude	1.27 (1.53)	4.14 (2.80)	15.94^b^	0.01*	0.48
Verbal Aggression	4.47 (3.09)	7.43 (2.64)	4.56^b^	0.05	0.21
Physical Aggression	0.80 (1.37)	1.43 (1.62)	2.06^b^	0.17	0.11

Note: Values are Mean (Standard Deviation). Statistics are from separate general linear models on the BPAQ and PAI, controlling for age, gender and depression. ^a^df(1,21); ^b^df(1,17); η^2^ = partial eta squared; ^‡^Small effect size: 0.01 ≤ η^2^ < 0.05; medium effect size: 0.06 ≤ η^2^ < 0.13; large effect size: η^2^ ≥ 0.14. mTBI, mild traumatic brain injury; BPAQ, Buss-Perry Aggression Questionnaire; PAI, Personality Assessment Inventory. Unadjusted p-values reported. *Significant at FDR-corrected p < 0.05.

### Neuroanatomical

The microstructure of targeted fiber pathways was compared between mTBI and HCs. In the corpus callosum, individuals with mTBI exhibited higher RD compared to HCs (*F*_(1, 21)_ = 7.71, *p* < 0.05; *η*^2^ = 0.27; FDR-corrected), indicating reduced fiber integrity within the corpus callosum after an mTBI. Lower FA in the corpus callosum was also found in the mTBI group, however this finding did not survive FDR-correction. White matter integrity (FA, MD, RD and AD) within the anterior thalamic radiation, cingulum, and uncinate fasciculus showed no significant between-group differences (see Supplementary Table S1).

### Neural Correlates of Aggression

**Figure 5:**
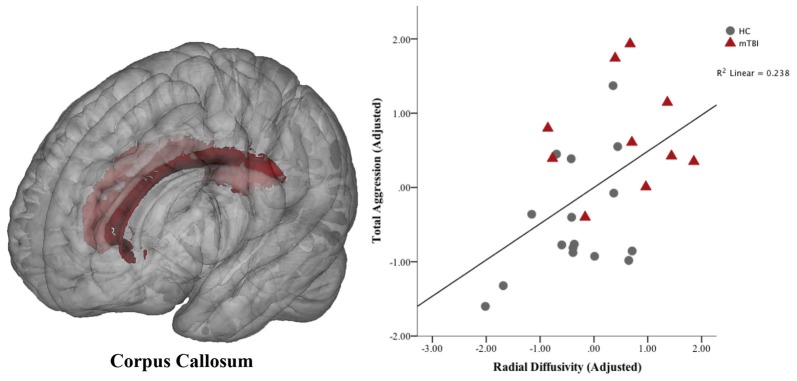
Association between aggression and white matter in the corpus callosum. Voxels of the corpus callosum included in the analysis are shown in red overlaid on a standard brain. Means are the standardized residuals after adjusting for covariates (age, gender and depression). A significant partial correlation (controlling for age, gender and depression) was found between total aggression on the Buss-Perry Aggression Questionnaire and radial diffusivity (RD) in the corpus callosum (*p* < 0.05; FDR-corrected). HC, healthy controls; mTBI, mild traumatic brain injury.

Neural correlates of aggression were restricted to overall aggression measures and fiber pathways that showed significant between-group differences in the previous analyses. Therefore, we assessed the relationship between white matter integrity in the corpus callosum (RD) and aggression (BPAQ-total aggression and PAI-total aggression). A significant positive correlation was found between RD in the corpus callosum and BPAQ-total aggression (*r* = 0.49; *p* < 0.05; FDR-corrected; see Figure 5).

*Post hoc* analyses were conducted to determine whether findings related aggression in the corpus callosum were widespread, or restricted to specific regions. Therefore, the corpus callosum was subdivided into the genu, body and splenium and template masks were created using the procedures previously described in the Materials and Methods: “Targeted White Matter Tracts” section. Similar to previous GLMs, between-group comparisons of white matter integrity in the corpus callosum were calculated for the three subdivisions separately (controlling for age, gender, and depression). Results are summarized in Table 3. Significantly higher RD was found in the body and the splenium of the corpus callosum in adults with mTBI compared to HCs (*p* < 0.05, uncorrected). While not significant across the entire corpus callosum, FA was significantly lower in the splenium for those with mTBI (*p* < 0.05, uncorrected). Partial correlations (controlling for age, gender and depression) were then calculated to determine which aspects of aggression were associated with the body and the splenium of the corpus callosum. Given the exploratory nature of these analyses, uncorrected *p*-values are reported. Physical aggression was only associated with white matter integrity in the splenium, in that higher reports of physical aggression were significantly correlated with higher RD (*r* = 0.42, *p* < 0.05) and lower FA (*r* = −0.43, *p* < 0.05). In contrast, aggressive attitude was significantly correlated with higher RD in the body of the corpus callosum (*r* = 0.64, *p* < 0.01).

**Table 3:** Diffusion characteristics for the corpus callosum.

	Healthy Controls (*n* = 16)	Chronic mTBI (*n* = 10)	*F-statistic* df (1, 21)	*p*-value	*Partial* η^2‡^
CC					
FA	0.80456 (0.01415)	0.79500 (0.01073)	4.32	0.05	0.17
MD	0.00072 (0.00002)	0.00074 (0.00002)	2.76	0.11	0.12
RD	0.00027 (0.00002)	0.00028 (0.00002)	7.71	0.01*	0.27
AD	0.00162 (0.00007)	0.00164 (0.00003)	0.16	0.69	0.01
CC Genu					
FA	0.80530 (0.02682)	0.79903 (0.01414)	0.13	0.72	0.01
MD	0.00071 (0.00002)	0.00072 (0.00003)	1.31	0.27	0.06
RD	0.00026 (0.00003)	0.00028 (0.00002)	0.53	0.48	0.02
AD	0.00159 (0.00008)	0.00162 (0.00007)	0.45	0.51	0.02
CC Body					
FA	0.78165 (0.01648)	0.77022 (0.02114)	3.60	0.07	0.15
MD	0.00074 (0.00003)	0.00076 (0.00002)	2.64	0.12	0.11
RD	0.00029 (0.00002)	0.00032 (0.00003)	5.68	0.03	0.21
AD	0.00163 (0.00007)	0.00165 (0.00003)	0.16	0.69	0.01
CC Splenium					
FA	0.83502 (0.01310)	0.82553 (0.01385)	4.94	0.04	0.19
MD	0.00070 (0.00003)	0.00071 (0.00002)	1.20	0.29	0.05
RD	0.00023 (0.00002)	0.00025 (0.00002)	4.95	0.04	0.19
AD	0.00164 (0.00008)	0.00164 (0.00003)	0.00	1.00	0.00

Note: Mean (Standard Deviation) in mm^2^/s. Values extracted from overlapping voxels in the 4D skeletonized image and template mask, for each region of the corpus callosum. General linear models were calculated for each interhemispheric tract separately, controlling for age, gender and depression. df, degrees of freedom. ^‡^Small effect size: 0.01 ≤ *η*^2^ < 0.05; medium effect size: 0.06 ≤ *η*^2^ < 0.13; large effect size: *η*^2^ ≥ 0.14. mTBI, mild traumatic brain injury; FA, fractional anisotropy; MD, mean diffusivity; RD, radial diffusivity; AD, axial diffusivity; CC, corpus callosum. Unadjusted p-values reported. *Significant at FDR-corrected p < 0.05.

## Discussion

In this study, we investigated post-concussive aggression in individuals who were in the chronic stage of recovery from mTBI, as well as the neural correlates of aggression. We hypothesized that individuals with mTBI would exhibit higher aggression relative to HCs, and that white matter integrity in the corpus callosum, cingulum, anterior thalamic radiation and uncinate fasciculus would be reduced in the mTBI population. In addition, we hypothesized that white matter integrity in these tracts would be associated with aggression. In support of our first hypothesis, we found significantly elevated levels of aggression in the mTBI group. Partial support was found for our second hypothesis, in that microstructure differed between the two groups, but only in the corpus callosum. We found significantly higher RD in the corpus callosum of adults with mTBI, which is indicative of reduced white matter integrity. Furthermore, a significant positive correlation was found between aggression and RD of the corpus callosum.

Numerous studies have reported increased aggression following traumatic brain injury (Kim et al., 1999; Bailie et al., 2015; Epstein et al., 2016; Roy et al., 2017), yet few have restricted participant samples to chronic mTBI to investigate the presence of persistent and potentially long-lasting symptoms associated with brain injury. Consistent with a recent study by Epstein et al. (2016), we found increased physical aggression, anger, and total aggression on the BPAQ following mTBI. Additionally, measures of aggressive attitude and total aggression on the PAI were elevated in those with mTBI. While not a focus of the present study, we found interesting gender differences following mTBI. In our sample, males with mTBI reported higher levels of physical aggression compared to females with mTBI. While some studies report no relationship between gender and aggression (Baguley et al., 2006; Johansson et al., 2008), our findings are consistent with a recent study focused exclusively on mild TBI. McGlade et al. (2015) investigated sex differences associated with functional connectivity following mTBI, and found that males exhibited increased physical aggression, which was associated with decreased left orbitofrontal functional connectivity. These findings may have important theoretical and clinical implications regarding the manifestation of aggression in the chronic recovery phase.

The BPAQ-anger and PAI-aggressive attitude subscales assess an individual’s tendency to become easily frustrated or lose control of one’s temper. High endorsement of these constructs by those with mTBI might be due to difficulties with the generation or subsequent understanding of affective responses (Lindquist et al., 2012; Smith et al., 2017). For example, it may be that individuals struggle to appropriately understand the causes and/or emotional meaning of felt somatovisceral changes during an affective response, which would be expected to hinder effective regulation of such responses. Thus, incomplete conceptualization of negative affect may manifest in initial frustration. On the other hand, increased levels of BPAQ-physical aggression suggest those with mTBI might struggle more with top-down control processes, which are important for appropriately adjusting behavioral responses within a particular context; if so, greater levels of dysregulated aggression would also be expected. In healthy adults, physical aggression is often higher in males than females (Archer, 2004; Cleverley et al., 2012). Although we did not observe this relationship exclusively in our healthy control group, we did find elevated physical aggression in males compared to females in those with mTBI. Based on our results, it may be that mTBI exacerbates a pre-existing gender difference, leaving males particularly prone to dysregulated emotional responses. Together, the current findings suggest persistent and variable manifestations of dysregulated aggression and frustration, perhaps related to impaired affect generation, conceptualization, and top-down control processes in chronic mTBI.

From this study, we provide preliminary findings regarding possible loci of pathology within neural networks that plausibly contribute to affective processing. Reduced integrity in the corpus callosum has previously been reported in mTBI (Lipton et al., 2008; Lo et al., 2009; Sugiyama et al., 2009). In line with these previous studies, we found increased RD in the corpus callosum in adults with chronic mTBI. The diffusivity pattern of increased RD is proposed to reflect a loss of structural integrity, resulting from myelination abnormalities and/or reduced axonal density (Beaulieu, 2002; Song et al., 2002; Concha et al., 2009). The lack of observed significant between-group differences for other diffusivity measures, particularly FA, is unclear. It is important to note that FA in the corpus callosum was reduced in the mTBI group compared to HCs. Although these findings did not survive correction for multiple comparisons, this was possibly the result of low power within our relatively small sample size. Overall, our findings provide preliminary evidence of persistent and/or dynamic loss of structural integrity within the corpus callosum in those who have experienced a mild TBI, and are in the chronic stage of recovery.

A major aim of our study was to evaluate the relationship between neuropsychological function and structural integrity of white matter pathways. The association between aggression and white matter integrity offers several potential implications regarding the underlying systems responsible for affective states. Cortical networks connected by the corpus callosum are known to play an integral role in the generation, conceptualization/representation, and regulation of affective/emotional responses (Lindquist et al., 2012; Smith and Lane, 2015). For example, one interpretation could be that reduced white matter in the corpus callosum might disrupt one’s ability to integrate exteroceptive and interoceptive percepts with conceptualization processes during an emotional episode, potentially contributing to situationally inappropriate aggressive responses and a poor conceptual understanding of those responses (i.e., low emotional awareness or alexithymia)—both of which would be expected to contribute to sustained dysregulation (Paul, 2004; Kubota et al., 2012). Considering previous findings implicating the corpus callosum (Kubota et al., 2012), as well as the interconnected cortical regions (Kalisch et al., 2006) in emotional awareness (or the related construct of alexithymia), our results suggest disruption in such cognitive-emotional processes might contribute to frustration or agitation in individuals with mTBI. This interpretation is also consistent with our *post hoc* analyses indicating reduced white matter integrity in the body and splenium of the corpus callosum, which have been associated with sensorimotor and perception/conceptualization processes, respectively (Paul, 2011).

An alternative interpretation of our findings could be that reduced white matter integrity in the corpus callosum results in decreased behavioral inhibition. Prior studies have documented increased aggression in patients with orbitofrontal damage (Coccaro et al., 2007; Epstein et al., 2016). Our findings indicate similar behavioral tendencies (increased aggression) associated with reduced structural integrity in the corpus callosum, which may disrupt interhemispheric signal transfer and impede cortically mediated inhibitory processes necessary for context-appropriate behavioral regulation. The different mechanistic interpretations we have proposed could also interact, such that difficulty integrating internal and external cues during conceptualization, combined with a lack of inhibitory control during the regulatory process, could result in the selection of inappropriate behaviors such as physical or verbal aggression. It should be mentioned, however, that disruptions within frontal (genu) subregions of the corpus callosum would be most consistent with deficits in inhibitory control; thus, the fact that alterations within posterior subregions of the corpus callosum appear to drive our findings, this is probably most consistent with deficits in recognizing and understanding one’s own emotions. Regardless of the detailed mechanisms involved, our findings suggest that alterations in the corpus callosum may play an important role in promoting dysregulated affect, leading to the observed increases in aggressive tendencies.

### Limitations and Future Directions

It is important to acknowledge methodological limitations of the current study. First, we assessed aggression at a single point in time (i.e., during the chronic stage of recovery). As such, we are unable to determine whether aggression manifests differently throughout various stages of recovery. For example, those in the acute phase might exhibit more outward aggression (i.e., physical or verbal aggression) as a result of reduced executive control, while those in the chronic phase might have relearned aspects of executive function, gaining more control over behavioral inhibition but continue to exhibit deficits with the context-appropriate generation or conceptualization of affective responses. While we are unable to answer this particular question about the role of different mechanistic contributions at different recovery stages, our current findings provide new insights into the manifestation of aggression in chronic mTBI.

We acknowledge the relatively small sample size of the present study. Although we implemented methodological approaches aimed at minimizing small sample effects (i.e., partial volume effects and targeted pathways), we may have lacked the statistical power necessary to detect group-differences in certain tracts. This is especially problematic for anatomical regions with a considerable number of crossing fibers (i.e., the uncinate fasciculus), as crossing fibers are another source of inherent noise in DTI studies (Jeurissen et al., 2013). Furthermore, the link between aggression and white matter integrity was calculated across all participants. As such, a disease-specific effect of brain structure on aggression cannot be inferred. Future studies with larger sample sizes are needed to confirm our preliminary findings and further examine the reliability of the reported relationship between aggression and fiber tracts in systems contributing to affective processing.

Limitations notwithstanding, the clinical implications of this study should not be overlooked. Aggressive behavior can have devastating impacts on the home environment, social and interpersonal functioning, and could result in the loss of employment or criminal violence. Therefore, therapeutic interventions aimed at ways to more effectively manage affective states may be particularly beneficial to individuals who experience persistent affective symptoms. Understanding the association between neural systems and behavior could potentially lead to interventions targeting the range of emotion-related processes that might be particularly affected by damage sustained from mTBI.

## Conclusion

In conclusion, we found higher levels of aggression in adults with a chronic mTBI, when compared to HCs. Additionally, elevated aggression was associated with reduced white matter integrity in the corpus callosum, regardless of group. As this pathway is known to play an important role in multiple affective processes, our results suggest that alterations in this tract may play an important role in accounting for dysregulated aggression. This may be associated with the generation of situationally inappropriate affective responses and/or poor awareness/understanding of such responses (i.e., both linked to disrupted conceptualization processes). Alternatively, it could be directly associated with reduced top-down cognitive/behavioral regulatory processes. Future research should focus on disambiguating which of these processes, or what combination of them, best accounts for such mTBI symptoms. This may be especially important, given that our findings also highlight the potentially persistent nature of such post-concussive symptoms in mTBI.

## Author Contributions

ND assisted with MRI data acquisition, conducted MRI data processing, statistical analysis and drafted the initial manuscript. RS and SB assisted with data analysis and interpretations, and manuscript revisions. AA assisted with the statistical analysis and manuscript revisions. MG conducted participant recruitment, data acquisition, MRI preprocessing and assisted with manuscript revisions. AR assisted with data interpretation and manuscript revisions. BS assisted with manuscript revisions. WK designed the study, assisted with data interpretation and critique, as well as manuscript review and revisions.

## Conflict of Interest Statement

The authors declare that the research was conducted in the absence of any commercial or financial relationships that could be construed as a potential conflict of interest.
