# Supplementary material for: Elevated Aggression and Reduced White Matter Integrity in Mild Traumatic Brain Injury: A DTI Study
Source: Front Behav Neurosci. 2018 Jun 27;12:118. doi: 10.3389/fnbeh.2018.00118 (PMC6036267; doi:10.3389/fnbeh.2018.00118)
Supplement: Supplementary file 1 [file Table_1.docx]

Supplementary Table

**Table S1.** Diffusion characteristics for intra-hemispheric tracts.

|  | | Healthy Controls  (n = 16) | Chronic mTBI  (n = 10) | *F-statistic*  df (1, 21) | *p*-value | *Partial* η^2‡^ |
| --- | --- | --- | --- | --- | --- | --- |
| ATR RH | |  |  |  |  |  |
| FA | 0.52695 (0.02282) | 0.50944 (0.01944) | 5.62 | 0.03 | 0.21 |  |
| MD | 0.00074 (0.00002) | 0.00075 (0.00002) | 1.13 | 0.30 | 0.05 |  |
| RD | 0.00050 (0.00002) | 0.00052 (0.00001) | 5.62 | 0.03 | 0.21 |  |
| AD | 0.00120 (0.00005) | 0.00120 (0.00005) | 0.22 | 0.64 | 0.01 |  |
| ATR LH | |  |  |  |  |  |
| FA | 0.52626 (0.02027) | 0.51188 (0.02305) | 4.31 | 0.05 | 0.17 |  |
| MD | 0.00073 (0.00003) | 0.00075 (0.00002) | 2.77 | 0.11 | 0.12 |  |
| RD | 0.00050 (0.00003) | 0.00052 (0.00002) | 5.65 | 0.03 | 0.21 |  |
| AD | 0.00121 (0.00005) | 0.00123 (0.00005) | 0.13 | 0.72 | 0.01 |  |
| Cing RH | |  |  |  |  |  |
| FA | 0.65534 (0.04136) | 0.64010 (0.02456) | 1.89 | 0.18 | 0.08 |  |
| MD | 0.00074 (0.00003) | 0.00076 (0.00002) | 2.70 | 0.12 | 0.11 |  |
| RD | 0.00041 (0.00004) | 0.00043 (0.00002) | 3.69 | 0.07 | 0.15 |  |
| AD | 0.00141 (0.00008) | 0.00143 (0.00005) | 0.01 | 0.93 | 0.01 |  |
| Cing LH | |  |  |  |  |  |
| FA | 0.69992 (0.03084) | 0.68807 (0.02415) | 1.14 | 0.30 | 0.05 |  |
| MD | 0.00075 (0.00002) | 0.00076 (0.00002) | 2.94 | 0.10 | 0.12 |  |
| RD | 0.00038 (0.00003) | 0.00039 (0.00002) | 3.05 | 0.10 | 0.13 |  |
| AD | 0.00151 (0.00008) | 0.00150 (0.00007) | 0.09 | 0.76 | 0.01 |  |
| UF RH | |  |  |  |  |  |
| FA | 0.55835 (0.04383) | 0.56087 (0.04349) | 0.00 | 0.99 | 0.01 |  |
| MD | 0.00078 (0.00004) | 0.00079 (0.00004) | 0.10 | 0.76 | 0.01 |  |
| RD | 0.00050 (0.00004) | 0.00050 (0.00005) | 0.03 | 0.86 | 0.01 |  |
| AD | 0.00134 (0.00008) | 0.00135 (0.00006) | 0.10 | 0.76 | 0.01 |  |
| UF LH | |  |  |  |  |  |
| FA | 0.56690 (0.04953) | 0.55636 (0.04461) | 0.03 | 0.87 | 0.01 |  |
| MD | 0.00076 (0.00006) | 0.00077 (0.00006) | 1.18 | 0.29 | 0.05 |  |
| RD | 0.00048 (0.00006) | 0.00049 (0.00005) | 0.36 | 0.55 | 0.02 |  |
| AD | 0.00131 (0.00007) | 0.00131 (0.00011) | 1.76 | 0.20 | 0.08 |  |
| *Note: Mean (Standard Deviation) in mm^2^/s. Values extracted from overlapping voxels in the 4D skeletonized image and template mask, for each tract. General linear models were calculated for each intra-hemispheric tract separately, controlling for age, gender, and depression. df, degrees of freedom; ^‡^Small effect size: 0.01 < η^2^ < 0.05; medium effect size: .06 < η^2^ < 0.13; large effect size: η^2^ > 0.14. mTBI, mild traumatic brain injury; FA, fractional anisotropy; MD, mean diffusivity; RD, radial diffusivity; AD, axial diffusivity; ATR, anterior thalamic radiation; Cing, cingulum; UF, uncinate fasciculus; RH, right hemisphere; LH, left hemisphere.*  *Unadjusted p-values reported.* | | | | | | |
